# Prediction of Pathological Complete Response to Neoadjuvant Immunotherapy-Based Regimens for Non-Metastatic Colon and Rectal Cancer

**DOI:** 10.3390/jcm15208022

**Published:** 2026-10-16

**Authors:** Xiaoyan Huang, Meiling Ye, Zhikun Liang, Ling Yang, Weiming Huang, Li Qin, Jingwen Xie, Xiaoyan Li

**Affiliations:** 1 Department of Pharmacy, The Sixth Affiliated Hospital, Sun Yat-Sen University, Guangzhou 510655, China; huangxy278@mail.sysu.edu.cn (X.H.); yemling3@mail2.sysu.edu.cn (M.Y.); liangzhk9@mail.sysu.edu.cn (Z.L.); yangling27@mail.sysu.edu.cn (L.Y.); 2 Guangdong Provincial Key Laboratory of Colorectal and Pelvic Floor Diseases, The Sixth Affiliated Hospital, Sun Yat-Sen University, Guangzhou 510655, China; 3 Biomedical Innovation Center, The Sixth Affiliated Hospital, Sun Yat-Sen University, Guangzhou 510655, China; 4 School of Pharmaceutical Science, Sun Yat-Sen University, Guangzhou 510275, China; 5 School of Pharmaceutical Science, Guangdong Pharmaceutical University, Guangzhou 510006, China; hwm012@163.com

**Keywords:** pathological complete response, neoadjuvant immunotherapy, colon and rectal cancer, immune checkpoint inhibitors, nomogram

## Abstract

**Background**: Pathological complete response (pCR) after neoadjuvant immunotherapy is associated with improved prognosis in colon and rectal cancer. This study aimed to develop and validate a predictive nomogram for pCR in CRC patients receiving neoadjuvant immunotherapy. **Methods**: A total of 232 eligible CRC patients who received immune checkpoint inhibitors (ICIs) followed by surgical resection at the Sixth Affiliated Hospital of Sun Yat-sen University (January 2018–December 2024) were retrospectively included, with 162 in the training set and 70 in the validation set. Univariate and multivariate logistic regression analyses were performed to identify factors influencing pCR, and a nomogram was subsequently developed. The area under the receiver operating characteristic curve (AUC), calibration curves, and decision curve analysis (DCA) were used to evaluate the discrimination, calibration and clinical value of the nomogram. **Results**: The pCR rate in the entire cohort was 53.4%, with rates of 76.8% (73/95) and 37.2% (51/137) in the MSI-H/dMMR and MSS/pMMR subgroups, respectively. In multivariable analysis, MSI-H/dMMR status and pretreatment CEA level < 5 ng/mL were independent predictors of pCR and were used to construct the nomogram. The nomogram achieved AUCs of 0.741 in the training set and 0.730 in the validation set. Calibration curve analysis showed good agreement between predicted and actual pCR in both sets, and decision curve analysis demonstrated favorable net benefit. **Conclusions**: Our nomogram may predict pCR and help identify likely responders to neoadjuvant immunotherapy-based regimens in non-metastatic CRC patients. Prospective multi-center validation is warranted to confirm its clinical utility.

## 1. Introduction

Colon and rectal cancer (CRC) is a major global health burden, ranking as the third most common cancer and the second leading cause of cancer-related mortality. The 2022 global estimates of over 1.9 million new cases and approximately 900,000 fatalities underscore its ongoing and substantial impact [1]. Neoadjuvant chemoradiotherapy (nCRT) is a cornerstone of CRC management, as it downstages tumors, increases radical resection and pathological complete response (pCR) rates, enables organ preservation, and guides subsequent treatment strategies [2]. Moreover, achieving a pCR after nCRT serves as a significant prognostic indicator and is associated with a markedly reduced risk of recurrence and improved long-term survival. However, with nCRT, only approximately 6.6–27.5% of patients with locally advanced CRC achieve pCR [3,4].

In recent years, major advancements have been maded in neoadjuvant immunotherapy for CRC, particularly in patients with microsatellite instability-high/mismatch repair deficiency (MSI-H/dMMR). In the NICHE and NICHE-2 studies, neoadjuvant nivolumab plus ipilimumab achieved pCR rates of 60% and 68%, respectively. In the NICHE-3 study, neoadjuvant nivolumab plus relatlimab achieved a 68% pCR rate, substantially surpassing that of traditional neoadjuvant chemoradiotherapy [5,6,7]. In microsatellite stable/mismatch repair proficient (MSS/pMMR) patients, the combination of nCRT and immunotherapy is associated with a significant increase in pCR [8,9,10]. In the UNION trial, short-course radiotherapy followed by camrelizumab and chemotherapy (CAPOX) achieved a pCR rate of 39.8% in locally advanced rectal cancer [10]. In the SPRING-01 trial, short-course radiotherapy followed by sintilimab and chemotherapy (CAPOX) achieved a pCR rate of 59.2% in locally advanced rectal cancer, which was significantly superior to the 32.7% observed in the CAPOX alone group [11]. However, neoadjuvant immunotherapy is not universally beneficial, even in some MSI-H/dMMR patients who still experience primary resistance or insufficient therapeutic response [12]. Moreover, radiological assessment alone cannot reliably evaluate treatment efficacy. A meta-analysis demonstrated that the concordance between imaging and pathological response in MSI-H/dMMR CRC cancer is merely 59.6%, with 64.2% of radiological assessments in colon cancer patients underestimating actual pathological regression [13]. Liao et al. reported that only 7.1% of pCR tumors achieved a radiological CR in dMMR colon cancer following neoadjuvant immunotherapy [14]. Additionally, immunotherapy may cause immune-related adverse events (irAEs), resulting in additional morbidity [15]. Therefore, accurate identification of responders and precise prediction of pathological complete response are essential to avoid ineffective treatment and associated toxicities, and to facilitate risk-stratified management and organ preservation strategies.

Currently, most established predictive models for pathological response have been developed for patients receiving neoadjuvant chemoradiotherapy, especially those with locally advanced rectal cancer [16,17]. By contrast, prediction tools in the neoadjuvant immunotherapy setting are still emerging and often rely on exploratory biomarkers, which may limit routine clinical applicability [18,19]. Potential predictors span clinicopathological characteristics (e.g., tumor stage and CEA levels), molecular biomarkers (e.g., MSI/MMR status), and treatment regimens [20,21]. However, whether these routinely available variables can be integrated into a practical prediction model for pCR in this setting remains unclear. Although MSI-H/dMMR and MSS/pMMR tumors differ fundamentally in biological behaviour and immune responsiveness, pCR is a clinically meaningful endpoint of neoadjuvant immunotherapy in both populations. Notably, MSI/MMR status is itself included among the candidate variables. An integrated model may therefore retain subtype-specific information while meeting the real-world need for a one-stop risk assessment. In this study, we therefore aimed to develop and validate a nomogram for predicting pCR in non-metastatic CRC patients receiving neoadjuvant immunotherapy-based regimens.

## 2. Materials and Methods

### 2.1. Study Population

CRC patients who underwent surgical resection after neoadjuvant immunotherapy at the Sixth Affiliated Hospital of Sun Yat-sen University between January 2018 and December 2024 were included in this study. Neoadjuvant immunotherapy was defined as the administration of at least one cycle of an immune checkpoint inhibitor (ICI) before surgery, including PD-1, PD-L1, CTLA-4 inhibitors, or bispecific antibodies. The inclusion criteria were as follows: (a) pathologically confirmed CRC; (b) receipt of at least one cycle of ICI-based neoadjuvant therapy; and (c) undergoing resection of the primary tumor. The exclusion criteria were as follows: (a) previous or concurrent other primary malignancies; (b) recurrent CRC; (c) distant metastasis before treatment; and (d) incomplete key clinical and pathological data. Eligible patients were enrolled and randomly allocated to the training cohort or the validation cohort at a 7:3 ratio using R software (R version 4.5.3) with a fixed random seed of 7. This study was approved by the Ethics Committee of the Sixth Affiliated Hospital of Sun Yat-sen University (protocol code: E2024045, date of approval: 4 February 2024) and was conducted in accordance with the principles of the Declaration of Helsinki. Owing to the retrospective nature of the study, informed consent was waived.

### 2.2. Data Collection and Study Assessments

Clinical data were retrospectively collected from electronic medical records. The extracted variables mainly included patient demographics (e.g., age, sex, and BMI), medical history (including family history of cancer, abdominal surgery, and comorbidities), key serum tumor markers (CEA, CA19-9, and CA125), and tumor characteristics such as pathology, primary tumor location, clinical stage, differentiation grade, and MSI/MMR status. MSI/MMR status was determined by immunohistochemistry (IHC) for MMR proteins (MLH1, PMS2, MSH2, MSH6) or PCR-based microsatellite analysis. Discordant cases were resolved by repeat testing, NGS (when available), or expert multidisciplinary consensus [22,23]. In addition, details of all therapeutic interventions, including radiotherapy, chemotherapy, targeted therapy, and immunotherapy, were also documented.

The tumor regression grade (TRG) was evaluated according to the NCCN guidelines. pCR was defined as the absence of residual tumor lesions in any excised tissue or lymph node, corresponding to TRG 0 and ypT0N0. All specimens were evaluated by experienced gastrointestinal pathologists independently, as part of routine clinical practice, according to standard criteria. Treatment-related adverse events (TRAEs) were recorded according to the National Cancer Institute Common Terminology Criteria for Adverse Events (CTCAE) version 5.0.

### 2.3. Statistical Analysis

We used SPSS 19.0 (IBM Corp., Armonk, NY, USA) and R version 4.5.3 (R Foundation for Statistical Computing, Vienna, Austria) to perform the statistical analysis. Continuous data were expressed as mean ± SD or median (IQR) and were compared using the Student’s *t*-test or Mann-Whitney U test. Categorical data were expressed as number (%) and were compared with the chi-square test or Fisher’s exact test. Predictors for pCR were identified using univariate logistic regression, with significant ones included in the multivariate analysis using the enter method. Before multivariate model construction, multicollinearity among candidate variables was assessed using the variance inflation factor (VIF). A VIF > 5 was considered indicative of potentially problematic multicollinearity. All variables had a VIF < 5 and were therefore entered into the multivariate analysis. Based on the independent predictors derived from the training cohort, a nomogram was developed. Bootstrap internal validation with 1000 iterations was performed to correct for overfitting, estimate optimism, and obtain optimism-corrected performance estimates. The performance of this model was then comprehensively evaluated across both the training and validation cohorts. Discriminative ability was assessed by plotting receiver operating characteristic (ROC) curves, while its calibration was evaluated using calibration curves and the Hosmer-Lemeshow goodness-of-fit test. The clinical utility of the nomogram was further examined via decision curve analysis (DCA). Statistical significance was defined as a two-sided *p* < 0.05.

## 3. Results

### 3.1. Baseline Characteristics of the Training and Validation Sets

**Figure 1:**
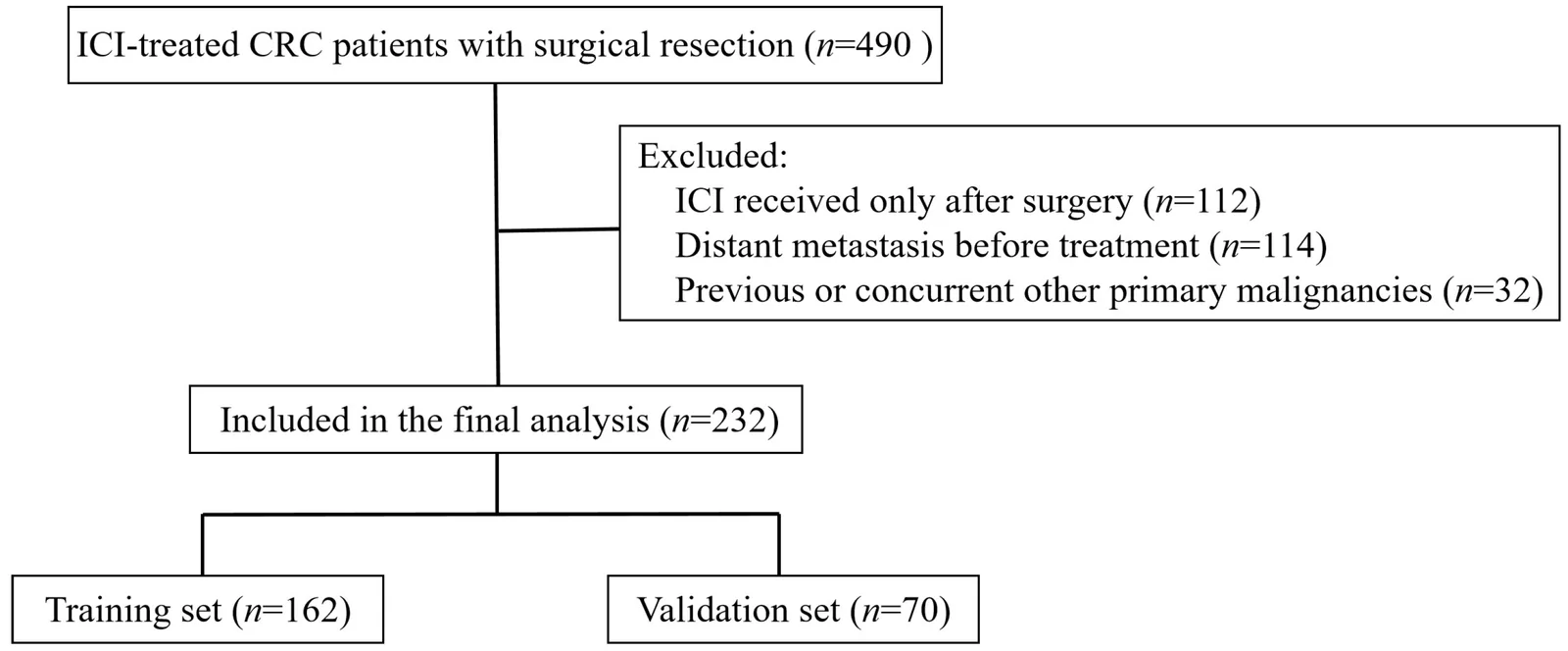
Flow chart of patient selection.

Between January 2018 and December 2024, 490 CRC patients who underwent ICI treatment and bowel resection were screened, and a total of 232 eligible patients with stage I–III CRC were included in this study (Figure 1), comprising 128 with rectal cancer and 104 with colon cancer. The ICIs used in this study included PD-1 inhibitors (toripalimab, sintilimab, pembrolizumab, and tislelizumab), a PD-L1 inhibitor (atezolizumab), and a bispecific PD-1/CTLA-4 antibody (cadonilimab). The distribution of neoadjuvant immunotherapy-based regimens was as follows: immunotherapy monotherapy (IO; *n* = 66), immunotherapy combined with chemotherapy (IO + CT; *n* = 44), triplet therapy combining immunotherapy, chemotherapy, and targeted therapy (IO + CT + TT; *n* = 58), and triplet therapy combining immunotherapy, chemotherapy, and radiotherapy (IO + CT + RT; *n* = 64). Among these eligible patients, 124 (53.4%) and 137 (59.1%) achieved pCR and major pathological response (MPR), respectively, after neoadjuvant immunotherapy. Among the 95 patients with MSI-H/dMMR, 73 achieved pCR (76.8%) and 78 achieved MPR (82.1%). In contrast, among the 137 patients with MSS/pMMR, 51 (37.2%) achieved pCR and 59 (43.1%) achieved MPR.

The included patients were randomly divided into a training set (*n* = 162) and a validation set (*n* = 70). Baseline characteristics of the two sets are summarized in Table 1. The pCR rate was 53.1% (86/162) in the training set and 54.3% (38/70) in the validation set. No significant differences in demographic medical history or tumor characteristics were observed between the two sets. Similarly, pathological features and therapeutic interventions were well balanced.

### 3.2. Adverse Events

The neoadjuvant TRAEs are presented in Table 2. All 232 patients reported at least one TRAE. Hypoproteinemia (94.0%), electrolyte disorders (94.4%), myelosuppression (84.9%), anemia (74.1%), leucopenia (46.6%), neutropenia (44.4%), and elevated serum ALT/AST (23.3%) were the most common TRAEs. Grade ≥ 3 TRAEs occurred in 98 (42.2%) patients, with no toxicity-induced death recorded. With the exception of infection (IO vs. IO + CT) and electrolyte disorders (IO vs. IO + CT + RT), no significant differences in Grade ≥ 3 TRAE profiles were observed across the four treatment regimens: IO, IO + CT, IO + CT + TT, and IO + CT + RT.

### 3.3. Univariate and Multivariate Logistic Analyses of the Training Set

The results of univariate and multivariate logistic regression in the training set (n = 162; 86 pCR events) are shown in Table 3. 22 candidate variables were assessed in univariable analysis. Of these, pretreatment CEA level, primary tumor location, microsatellite status, targeted therapy course, and chemotherapy course were significantly associated with pCR (*p* < 0.05) and were therefore entered into the multivariable analysis. In multivariable analysis, the pretreatment CEA level (OR = 0.390; 95% CI: 0.189–0.803; *p* = 0.011) and MSI/MMR status (OR = 5.151; 95% CI: 1.092–24.301; *p* = 0.038) were the only variables identified as independent predictors significantly associated with pCR.

### 3.4. Nomogram for Predicting pCR Probability

**Figure 2:**
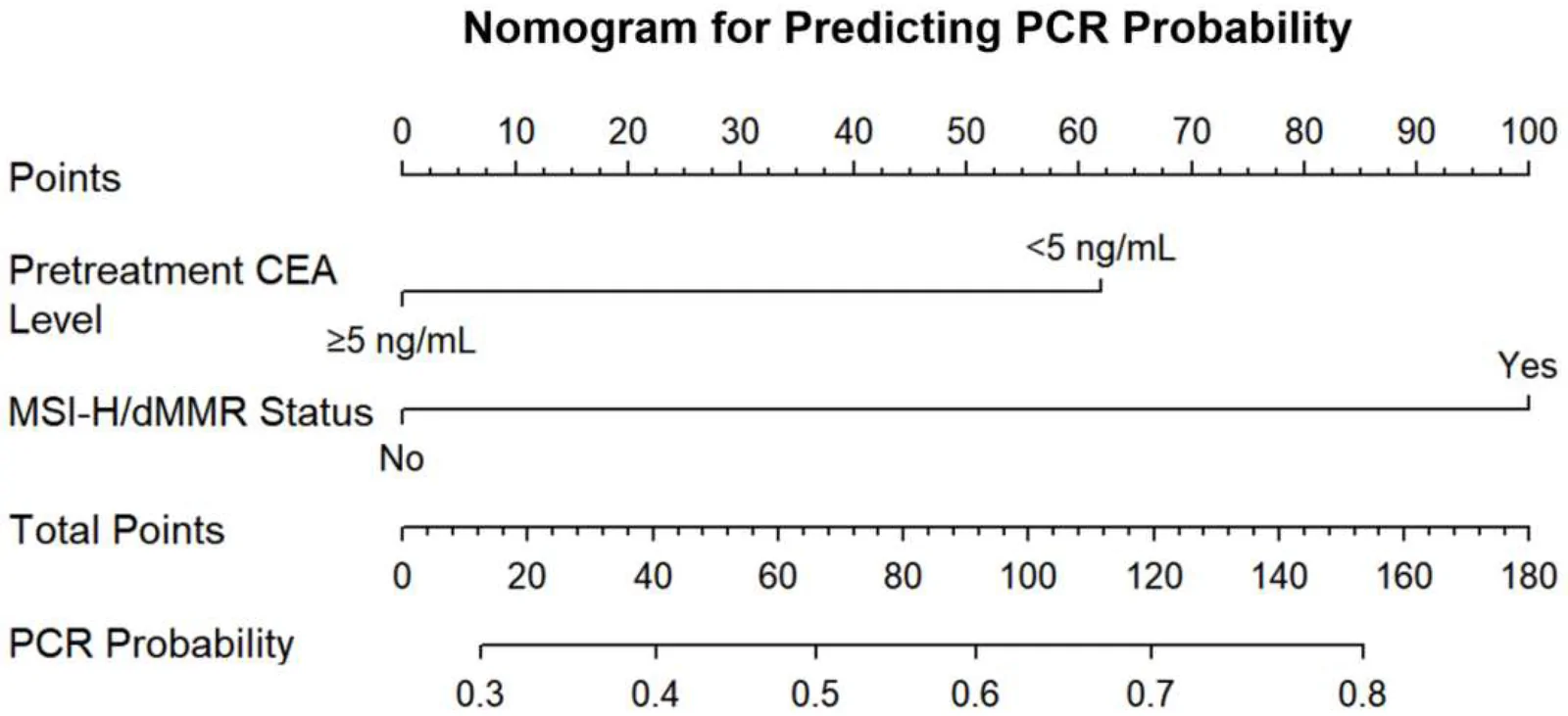
The training set based nomogram for predicting the probability of pCR in CRC patients receiving neoadjuvant immunotherapy-based regimens.

Using the independent predictors derived from the multivariate logistic regression analysis, we constructed a predictive nomogram for pCR (Figure 2). Each variable was converted into a weighted score on the nomogram. The total score, calculated as the sum of these individual scores, corresponded directly to a predicted probability of pCR.

### 3.5. Model Performance and Validation

**Figure 3:**
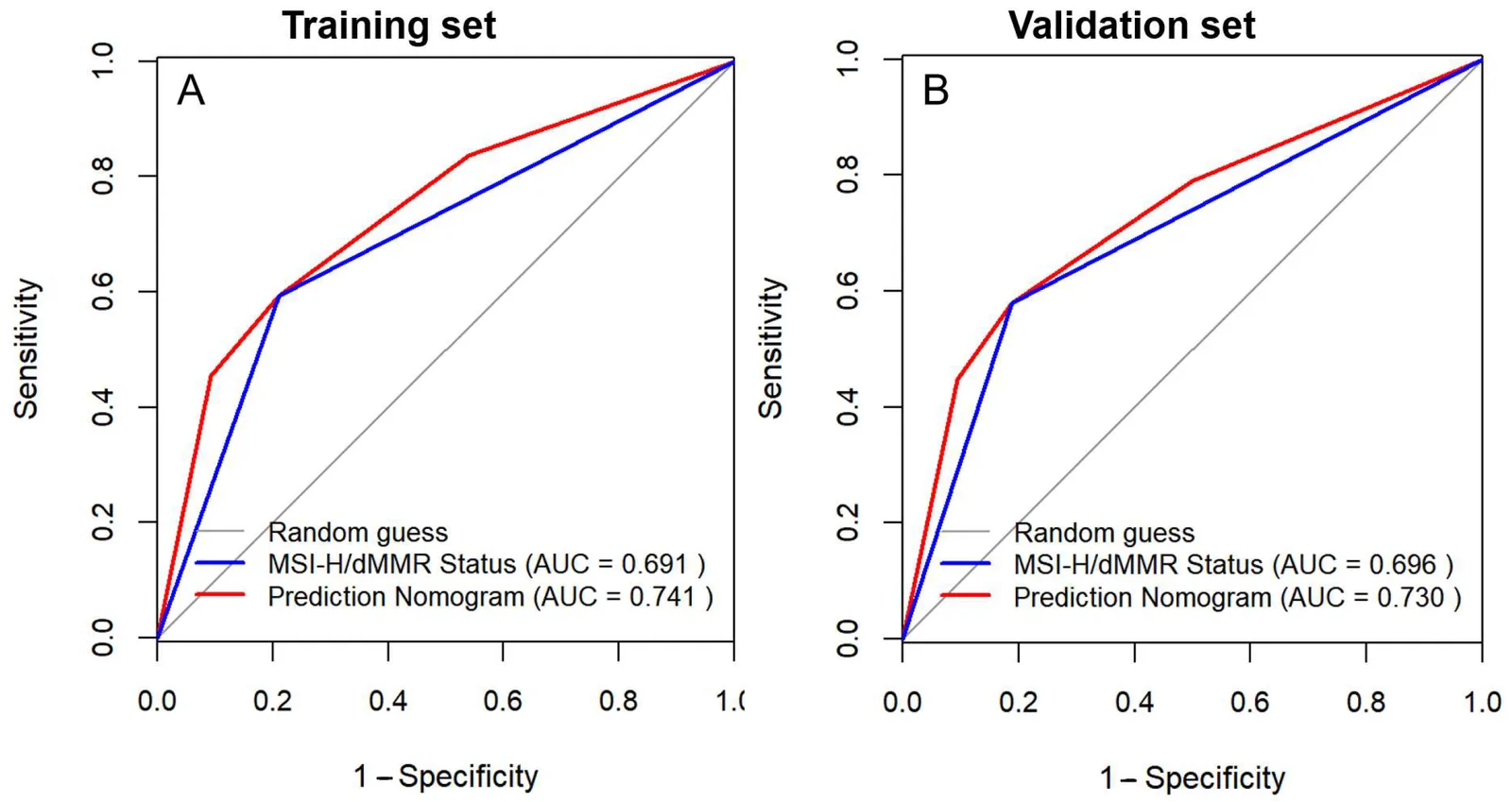
The ROC curves of the nomogram model in the training set (**A**) and validation set (**B**).

**Figure 4:**
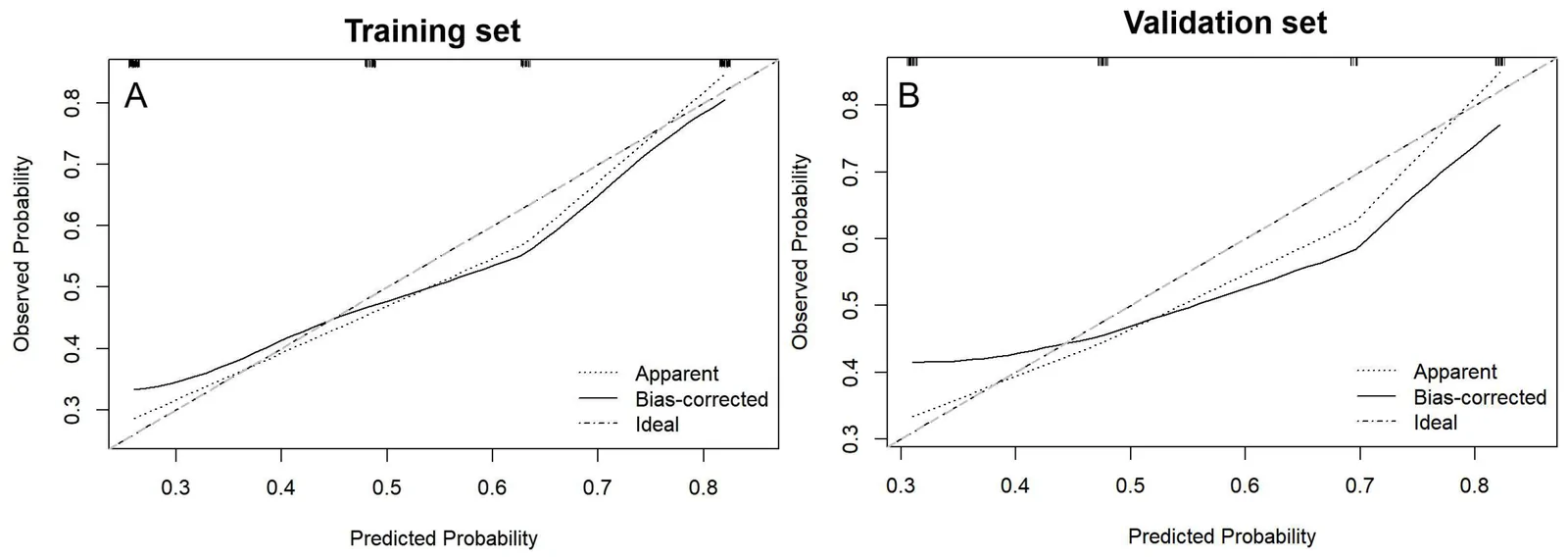
The calibration curves for the nomogram model in the training set (**A**) and validation set (**B**).

**Figure 5:**
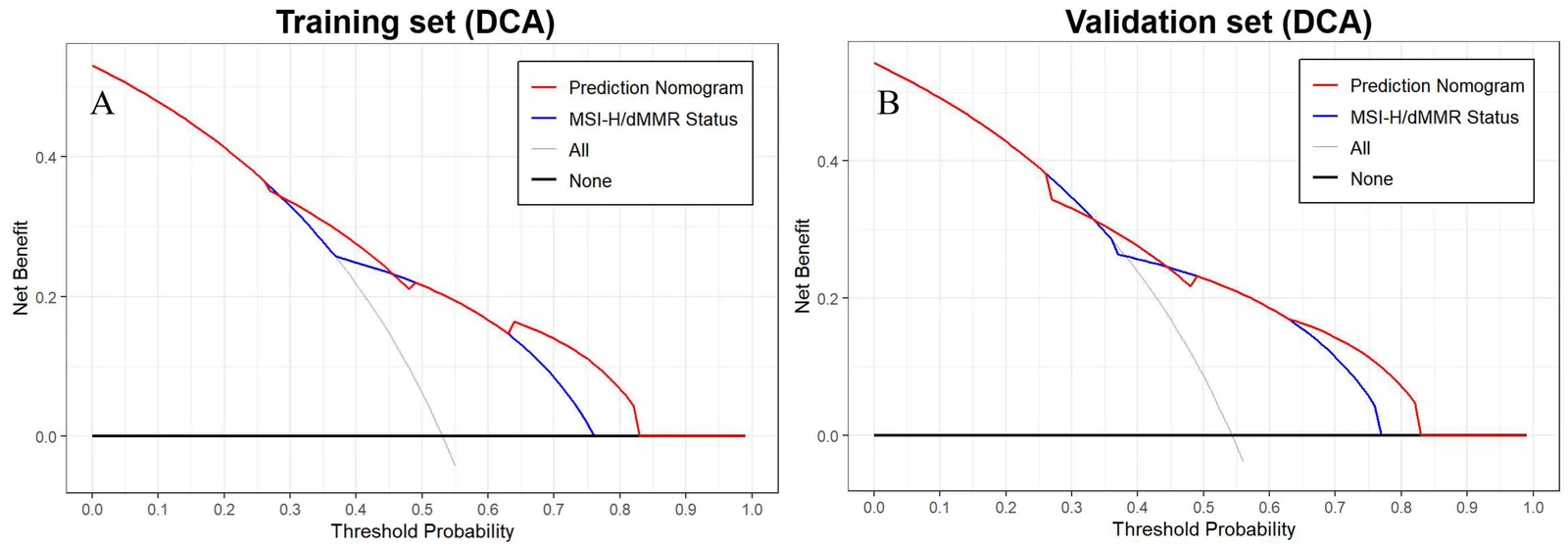
The decision curves for the nomogram in the training set (**A**) and validation cohort (**B**).

Figure 3 presents the ROC curves of the nomogram in the training set (Figure 3A) and the validation set (Figure 3B). The areas under the receiver operating characteristic curve (AUC) of MSI-H/dMMR status alone were 0.691 (95% CI: 0.622–0.761) in the training set and 0.696 (95% CI: 0.591–0.801) in the validation set. After incorporating pretreatment CEA level, the nomogram achieved an AUC of 0.741 (95% CI: 0.668–0.814) in the training set and 0.730 (95% CI: 0.616–0.844) in the validation set, demonstrating that adding CEA improved predictive performance beyond MSI-H/dMMR status alone. The nomogram showed favorable predictive performance, with good discrimination evidenced by the AUC values and acceptable calibration in both sets. Bootstrap internal validation with 1000 resamples demonstrated that the nomogram had an apparent AUC of 0.741 and an optimism-corrected AUC of 0.734, with an optimism of 0.007, indicating minimal overfitting. The calibration curves are shown in Figure 4, with an optimism-corrected calibration intercept of −0.004 in the training set and −0.005 in the validation set (ideal value: 0), and a calibration slope of 0.975 in the training set and 0.940 in the validation set (ideal value: 1), indicating acceptable calibration with minimal overfitting. The Brier score was 0.202 in the training set and 0.204 in the validation set, indicating acceptable overall predictive accuracy. Hosmer-Lemeshow test indicated a good fit for the model, with no significant deviation from perfect calibration observed (training set: χ^2^ = 0.864, *p* = 0.649; validation set: χ^2^ = 3.845, *p* = 0.279). Additionally, DCA affirmed the clinical utility of the nomogram, as a significant net benefit was observed over a broad spectrum of threshold probabilities (Figure 5).

## 4. Discussion

In this study, 53.4% of CRC patients who received neoadjuvant immunotherapy-based regimens achieved pCR, with rates of 76.8% and 37.2% in the MSI-H/dMMR and MSS/pMMR subgroups, respectively. Previous studies reported that patients who received conventional chemotherapy alone had a pCR rate of approximately 10%; when radiotherapy was combined with chemotherapy, the pCR rate increased to 15–30% [4,24,25]. Furthermore, studies have shown that combining chemotherapy with targeted therapy can achieve a pCR rate of approximately 13–35% [26,27,28]. Neoadjuvant immunotherapy has shown promising efficacy in CRC. pCR rates range from 25% to 79% for ICIs, with 50–79% for MSI-H tumors and 25–62.5% for MSS tumors [8,29,30,31]. Consistent with previous studies, our results also demonstrated that the pCR rate was significantly higher in CRC patients who received neoadjuvant immunotherapy than in those who received chemotherapy alone or combined with targeted therapy or radiotherapy, particularly in the MSI-H/dMMR subgroup. This enhanced sensitivity to immunotherapy is likely attributable to the high tumor mutational burden (TMB), abundant neoantigens, and POLE/POLD1 mutations [32,33,34]. However, response to immunotherapy is not uniform. Some MSI-H/dMMR patients still failed to achieve pCR, indicating response heterogeneity within this subgroup that may involve antigen presentation defects, IFN-γ signaling impairment, compensatory immune checkpoint activation, and Wnt/β-catenin dysregulation [23,35,36,37]. By contrast, MSS/pMMR CRC is generally considered refractory to immunotherapy, characterized by low TMB and reduced immunogenicity [38,39]. Although these biomarkers help explain the biological basis of treatment response, they are not routinely obtained in clinical practice.

Given this biological complexity and the resulting heterogeneity in treatment response, a reliable predictive tool is needed to guide clinical decision-making. Importantly, however, the variables that clinicians can actually measure before initiating ICI therapy remain limited. We therefore developed a predictive model based on these routinely available clinical variables to estimate the probability of achieving pCR following neoadjuvant immunotherapy in both MSS and MSI-H patients. In this model, pretreatment CEA level < 5 ng/mL and MSI-H/dMMR status were identified as independent predictors of pCR in CRC patients receiving neoadjuvant immunotherapy-based regimens.

A study by Le et al. demonstrated that dMMR can serve as a biomarker for predicting the response to PD-1 blockade therapy across multiple cancer types [40]. Consistently, our findings suggest that MSI/MMR status is a predictor of pCR in patients receiving neoadjuvant immunotherapy-based regimens. These findings further support MSI-H/dMMR status as an effective biomarker for identifying patients most likely to benefit from neoadjuvant immunotherapy. Current clinical guidelines also recommend that all newly diagnosed CRC patients undergo MMR protein immunohistochemistry or MSI testing.

CEA is a broad-spectrum tumor biomarker and is commonly used as a screening tool for malignancies, as well as for monitoring treatment response and disease recurrence [41]. Previous retrospective studies have reported a correlation between lower CEA levels and pCR in patients with rectal cancer [42,43]. Li et al. indicated that a preoperative CEA level ≤ 3.35 ng/mL may predict pCR following neoadjuvant therapy in rectal cancer patients [43]. Moreover, Huh et al. revealed that CRC patients with relatively low CEA levels had significantly better survival than those with high CEA levels [44]. Consistent with these findings, our study also indicated that CRC patients with CEA level < 5 ng/mL were more likely to achieve pCR than those with CEA level ≥ 5 ng/mL.

In this study, although colon and rectal cancers differ in anatomy and neoadjuvant treatment strategies, primary tumor site was not an independent predictor of pCR. In univariate analysis, right-sided tumors were significantly associated with pCR (OR 2.34); however, in multivariate analysis, this association vanished after MSI/MMR status, pretreatment CEA, and other variables were entered into the model. This may be because right-sided colon cancers are more frequently enriched with MSI-H/dMMR [45], and the strong effect of MSI-H/dMMR status on pCR may have absorbed the effect of tumor sidedness. Consistent with previous studies in CRC patients receiving neoadjuvant immunotherapy, tumor site was not significantly associated with pCR in either MSS/pMMR or mixed cohorts containing both MSI-H/dMMR and MSS/pMMR populations [20,21], and it was not an independent predictor in these studies. Similarly, targeted therapy and chemotherapy were significant univariate factors but lost significance in multivariate analysis. This may be because MSI-H/dMMR tumors are known to have limited responsiveness to conventional chemotherapy [46], and targeted therapy and combination regimens were mainly given to MSS/pMMR patients, whereas MSI-H patients preferentially received single-agent immunotherapy [47,48]. In summary, MSI-H/dMMR status, as a tumor-intrinsic biomarker reflecting fundamental tumor immunogenicity and neoantigen burden, appeared to determine the baseline susceptibility to immune checkpoint blockade. Lower preoperative CEA may reflect a lower residual tumor burden [49,50]. In this study, among MSI-H/dMMR patients with low CEA (<5 ng/mL), the observed pCR rate was 56/66 (84.8%), compared with 29/64 (45.3%) in MSS/pMMR patients with the same characteristics. These rates were higher than the overall pCR rates of 76.8% and 37.2% observed in the MSI-H/dMMR and MSS/pMMR subgroups, respectively. Several pCR prediction models have been proposed for CRC neoadjuvant immunotherapy, including a radiomics-based model developed in MSI-H/dMMR CRC (AUC 0.904, requiring RNA-seq) [51], a pathomics-based model developed in a mixed pMMR/dMMR CRC cohort (AUC 0.830) [52], and a multimodal model integrating dynamic imaging, endoscopic features, and biopsy results for dMMR colon cancer (AUC 0.900) [14]. In contrast, our nomogram relies on two routine clinical parameters, offering greater simplicity and broader applicability, and providing a non-invasive means of identifying CRC patients most likely to achieve pCR before immune checkpoint therapy. These findings may inform future hypothesis-generating studies on organ-preservation strategies, although prospective validation is warranted before any clinical application.

Our study had several limitations. First, as a single-center retrospective study, selection bias may exist. Second, the limited sample size and uneven temporal patient distribution precluded both MSI-stratified subgroup analysis and temporal validation. Consequently, the incremental clinical value of our pooled-cohort model across molecular subtypes remains to be clarified. Third, potential predictive factors, such as PD-L1 expression, TMB, and POLE/POLD1 gene status, were not included in the model, which may have reduced its predictive performance. Incorporating deeper biological insights, such as those revealed by recent studies on tumor metabolism, autophagy, and heterogeneity, may further optimize predictive models in the future [53,54,55,56]. Moreover, external validation in larger, prospective multi-center cohorts is warranted to confirm our findings, further optimize the model, and assess its temporal generalizability. Despite these limitations, the proposed nomogram for predicting pCR offers a visual tool for identifying potential responders among CRC patients, which may help inform personalized treatment decisions.

## 5. Conclusions

In conclusion, among the commonly accessible clinical variables, preoperative CEA level < 5 ng/mL and MSI-H/dMMR status were the only variables identified as independent predictors of pCR in non-metastatic CRC patients who received neoadjuvant immunotherapy-based regimens. The nomogram integrating these two variables showed potential for predicting pCR and may help identify patients likely to achieve pCR. Prospective multi-center validation is warranted to further confirm its clinical utility.

## Figures and Tables

**Table 1:** Baseline characteristics between training set and validation set.

Characteristics	Training Set (*n* = 162)	Validation Set (*n* = 70)	Statistic Value	*p*
Age, *n* (%)			3.069	0.080
<50 years	48 (29.6)	29 (41.4)		
≥50 years	114 (70.4)	41 (58.6)		
BMI			−0.240	0.811
<18.5 kg/m^2^	11 (6.8)	5 (7.1)		
18.5–24.9 kg/m^2^	124 (76.5)	52 (74.3)		
≥25 kg/m^2^	27 (16.7)	13 (18.6)		
Gender, *n* (%)			1.101	0.294
Female	51 (31.5)	27 (38.6)		
Male	111 (68.5)	43 (61.4)		
Comorbidity, *n* (%)			1.385	0.725
Cardiovascular disease	15 (9.3)	6 (8.6)		
Diabetes mellitus	11 (6.8)	3 (4.3)		
other	14 (8.6)	9 (12.9)		
Family history, *n* (%)	7 (4.3)	3 (4.3)	0.000	1.000
History of abdominal surgery, *n* (%)	20 (12.3)	9 (12.9)	0.012	0.914
cT stage, *n* (%)			−0.432	0.666
T1	0 (0)	1 (1.4)		
T2	3 (1.9)	1 (1.4)		
T3	52 (32.1)	20 (28.6)		
T4	103 (63.6)	48 (68.6)		
cN stage, *n* (%)			−0.011	0.991
N0	13 (8.0)	7 (10.0)		
N1	50 (30.9)	19 (27.1)		
N2	86 (53.1)	37 (52.9)		
cTNM stage, *n* (%)			−0.273	0.785
I	3 (1.9)	1 (1.4)		
II	11 (6.8)	6 (8.6)		
III	145 (89.5)	63 (90.0)		
Pretreatment CEA level, *n* (%)			0.124	0.724
<5 ng/mL	92 (56.8)	38 (54.3)		
≥5 ng/mL	70 (43.2)	32 (45.7)		
Pretreatment CA125 level, *n* (%)			0.053	0.819
<35 U/mL	138 (85.2)	62 (88.6)		
≥35 U/mL	15 (9.3)	6 (8.6)		
Pretreatment CA199 level, *n* (%)			2.016	0.156
<37 U/mL	130 (80.2)	52 (74.3)		
≥37 U/mL	24 (14.8)	16 (22.9)		
Primary tumor location, *n* (%)			0.004	0.948
Left tumor	121 (74.7)	52 (74.3)		
Right tumor	41 (25.3)	18 (25.7)		
Histological subtype			0.000	1.000
Adenocarcinoma	156 (96.3)	68 (97.1)		
Other	6 (3.7)	2 (2.9)		
Tumor differentiation, *n* (%)			−0.602	0.951
Well	12 (7.4)	4 (5.7)		
Moderate	108 (66.7)	45 (64.3)		
Poor	20 (12.3)	7 (10.0)		
MSI/MMR status, *n* (%)			0.037	0.847
MSS/pMMR	95 (58.6)	42 (60.0)		
MSI-H/dMMR	67 (41.4)	28 (40.0)		
Radiation therapy, *n* (%)			0.292	0.589
No	119 (73.5)	49 (70.0)		
Yes	43 (26.5)	21 (30.0)		
Chemotherapy course, *n* (%)			2.160	0.345
0	48 (29.6)	18 (25.7)		
>0~<4	16 (9.9)	4 (5.7)		
≥4	89 (54.3)	46 (65.7)		
ICI course, *n* (%)			0.471	0.492
<4	24 (14.8)	8 (11.4)		
≥4	138 (85.2)	62 (88.6)		
Source of ICIs, *n* (%)			0.530	0.467
Domestic	147 (90.7)	66 (94.3)		
Imported	10 (6.2)	2 (2.9)		
ICI type			0.036	1.000
PD1	152 (93.8)	66 (94.3)		
PDL1	4 (2.5)	2 (2.9)		
PD-1/CTLA-4	5 (3.1)	2 (2.9)		
Targeted therapy course, *n* (%)			0.514	0.861
0	118 (72.8)	52 (74.3)		
>0~<4	8 (2.9)	2 (2.9)		
≥4	36 (22.2)	16 (22.9)		
MPR, *n* (%)	97 (59.9)	43 (61.4)	0.049	0.824
pCR, *n* (%)	86 (53.1)	38 (54.3)	0.028	0.867

**Table 2:** **Neoadjuvant treatment-related adverse events (TRAEs)**.

Event	TRAEs, *n* (%)		IO (*n* = 66), *n* (%)		IO + CT (*n* = 44), *n* (%)		IO + CT + TT (*n* = 58), *n* (%)		IO + CT + RT (*n* = 64), *n* (%)		*p*
	Any	Grade ≥ 3	Any	Grade ≥ 3	Any	Grade ≥ 3	Any	Grade ≥ 3	Any	Grade ≥ 3	
Any TRAE	232 (100)	98 (42.2)	66 (100.0)	23 (34.8)	44 (100.0)	22 (50.0)	58 (100.0)	22 (37.9)	64 (100.0)	31 (48.4)	0.260
Skin reaction	19 (8.2)	5 (2.2)	2 (3.0)	0 (0.0)	4 (9.1)	1 (2.3)	2 (3.4)	2 (3.4)	10 (15.6)	2 (3.1)	0.515
Fatigue	6 (2.6)	0 (0.0)	0 (0.0)	0 (0.0)	2 (4.5)	0 (0.0)	2 (3.4)	0 (0.0)	2 (3.1)	0 (0.0)	-
Infection	7 (3)	7 (3)	0 (0.0)	0 (0.0) *	4 (9.1)	4 (9.1)	1 (1.7)	1 (1.7)	2 (3.1)	2 (3.1)	0.036
Numbness in the extremities	5 (2.2)	0 (0.0)	0 (0.0)	0 (0.0)	2 (4.5)	0 (0.0)	0 (0.0)	0 (0.0)	3 (4.7)	0 (0.0)	-
Fever	10 (4.3)	2 (0.9)	0 (0.0)	0 (0.0)	5 (11.4)	1 (2.3)	0 (0.0)	0 (0.0)	5 (7.8)	1 (1.6)	0.453
Diarrhea	6 (2.6)	0 (0.0)	0 (0.0)	0 (0.0)	2 (4.5)	0 (0.0)	2 (3.4)	0 (0.0)	2 (3.1)	0 (0.0)	-
Thyroid Dysfunction	4 (1.7)	0 (0.0)	1 (1.5)	0 (0.0)	2 (4.5)	0 (0.0)	1 (1.7)	0 (0.0)	0 (0.0)	0 (0.0)	-
Myelosuppression	197 (84.9)	35 (15.1)	58 (87.9)	20 (30.3)	36 (81.8)	14 (31.8)	46 (79.3)	17 (29.3)	57 (89.1)	23 (35.9)	0.864
Anemia	172 (74.1)	52 (22.4)	49 (74.2)	19 (28.8)	34 (77.3)	9 (20.5)	41 (70.7)	10 (17.2)	48 (75)	14 (21.9)	0.472
Leucopenia	108 (46.6)	9 (3.9)	26 (39.4)	1 (1.5)	19 (43.2)	3 (6.8)	26 (44.8)	3 (5.2)	37 (57.8)	2 (3.1)	0.455
Neutropenia	103 (44.4)	25 (10.8)	26 (39.4)	3 (4.5)	15 (34.1)	4 (9.1)	32 (55.2)	9 (15.5)	30 (46.9)	9 (14.1)	0.162
Thrombocytopenia	37 (15.9)	8 (3.4)	4 (6.1)	2 (3.0)	7 (15.9)	2 (4.5)	11 (19)	2 (3.4)	15 (23.4)	2 (3.1)	1.000
Venous Thromboembolism	1 (0.4)	0 (0.0)	0 (0.0)	0 (0.0)	0 (0.0)	0 (0.0)	0 (0.0)	0 (0.0)	1 (1.6)	0 (0.0)	-
Hypopituitarism	1 (0.4)	1 (0.4)	0 (0.0)	0 (0.0)	0 (0.0)	0 (0.0)	1 (1.7)	1 (1.7)	0 (0.0)	0 (0.0)	0.440
Electrolyte disorders	219 (94.4)	16 (6.9)	61 (92.4)	0 (0.0)	42 (95.5)	3 (6.8)	56 (96.6)	4 (6.9)	60 (93.8)	7 (10.9) *	0.031
Elevated ALT/AST	54 (23.3)	8 (3.4)	9 (13.6)	4 (6.1)	15 (34.1)	1 (2.3)	8 (13.8)	2 (3.4)	22 (34.4)	1 (1.6)	0.607
Elevated bilirubin	20 (8.6)	3 (1.3)	3 (4.5)	0 (0.0)	2 (4.5)	1 (2.3)	6 (10.3)	0 (0.0)	9 (14.1)	2 (3.1)	0.229
Elevated serum creatinine	6 (2.6)	2 (0.9)	5 (7.6)	1 (1.5)	2 (4.5)	1 (2.3)	2 (3.4)	0 (0.0)	0 (0.0)	0 (0.0)	0.561
Proteinuria	26 (11.2)	1 (0.4)	6 (9.1)	1 (1.5)	5 (11.4)	0 (0.0)	8 (13.8)	0 (0.0)	7 (10.9)	0 (0.0)	1.000
Hypoproteinemia	218 (94.0)	0 (0.0)	34 (51.5)	0 (0.0)	40 (90.9)	0 (0.0)	57 (98.3)	0 (0.0)	60 (93.8)	0 (0.0)	-

Treatment-related adverse events: TRAEs; Immunotherapy monotherapy: IO; Immunotherapy plus chemotherapy: IO + CT; Triplet Therapy of immunotherapy, chemotherapy, and targeted Therapy: IO + CT + TT; Triplet therapy combining immunotherapy, chemotherapy, and radiotherapy: IO + CT + RT. For grade ≥3 TRAEs, * *p* < 0.05 vs. IO; Values without superscripts indicate not significant.

**Table 3:** Uni- and multivariate logistic regression analysis for pathological complete response after neoadjuvant Immunotherapy in the training set.

Factor	Univariate Analysis			Multivariate Analysis		
	OR	95%CI	*p*	OR	95%CI	*p*
Age, *n* (%)	0.740	0.374–1.462	0.386			
<50 years						
≥50 years						
BMI	1.482	0.762–2.873	0.247			
<18.5 kg/m^2^						
18.5–24.9 kg/m^2^						
≥25 kg/m^2^						
Gender	0.899	0.462–1.749	0.754			
Female						
Male						
Comorbidity	1.058	0.764–1.466	0.733			
Cardiovascular disease						
Diabetes mellitus						
other						
Family history	0.651	0.141–3.004	0.582			
History of abdominal surgery	1.378	0.531–3.576	0.509			
cT stage	1.188	0.651–2.167	0.754			
T1						
T2						
T3						
T4						
cN stage	1.358	0.826–2.234	0.228			
N0						
N1						
N2						
cTNM stage	1.797	0.722–4.470	0.207			
I						
II						
III						
Pretreatment CEA level	0.315	0.165–0.602	<0.001	0.390	0.189–0.803	0.011
<5 ng/mL						
≥5 ng/mL						
Pretreatment CA125 level	1.833	0.596–5.643	0.291			
<35 U/mL						
≥35 U/mL						
Pretreatment CA199 level	1.238	0.513–2.989	0.635			
<37 U/mL						
≥37 U/mL						
Primary tumor location	2.340	1.107–4.944	0.026	1.096	0.415–2.893	0.853
Left tumor						
Right tumor						
Histological subtype	0.429	0.076–2.409	0.336			
Adenocarcinoma						
Other						
Tumor differentiation	0.978	0.487–1.968	0.951			
Well						
Moderate						
Poor						
MSI/MMR status	5.464	2.715–10.997	<0.001	5.151	1.092–24.301	0.038
MSS/pMMR						
MSI-H/dMMR						
Radiation therapy	0.614	0.304–1.240	0.174			
Chemotherapy course	0.447	0.301–0.662	<0.001	1.333	0.519–2.477	0.754
0						
>0~<4						
≥4						
ICI course	1.716	0.713–4.130	0.228			
<4						
≥4						
Source of ICIs, *n* (%)	0.838	0.233–3.016	0.786			
Domestic						
Imported						
ICI type	0.639	0.269–1.518	0.310			
PD1						
PDL1						
PD-1/CTLA-4						
Targeted therapy course	0.523	0.352–0.778	0.001	0.832	0.517–1.337	0.447
0						
>0~<4						
≥4						

## Data Availability

The anonymized patient datasets created and/or analysed for the current study are available from the corresponding author on reasonable request.
